# The Effect of Mind-Body Therapies on Insomnia: A Systematic Review and Meta-Analysis

**DOI:** 10.1155/2019/9359807

**Published:** 2019-02-13

**Authors:** Xiang Wang, Peihuan Li, Chen Pan, Lisha Dai, Yan Wu, Yunlong Deng

**Affiliations:** ^1^Department of Clinical Psychology, the Third Xiangya Hospital, Central South University, Changsha, Hunan, 410013, China; ^2^Psychosomatic Health Institute, the Third Xiangya Hospital, Central South University, Changsha, Hunan, 410013, China

## Abstract

**Background/Purpose:**

Sleep plays an important role in individuals' health. The functions of the brain, the cardiovascular system, the immune system, and the metabolic system are closely associated with sleep. As a prevalent sleep disorder, insomnia has been closely concerned, and it is necessary to find effective therapies. In recent years, a growing body of studies has shown that mind-body therapies (MBTs) can improve sleep quality and ameliorate insomnia severity. However, a comprehensive and overall systematic review has not been conducted. In order to examine the effect of MBTs on insomnia, we conducted a systematic review and meta-analysis evaluating the effects of MBTs on sleep quality in healthy adults and clinical populations.

**Methods:**

PubMed, EMBASE, the Cochrane Library, and review of references were searched up to July 2018. English language studies of all designs evaluating the effect of MBTs on sleep outcomes in adults with or without diseases were examined. To calculate the SMDs and 95% CIs, we used a fixed effect model when heterogeneity was negligible and a random effect model when heterogeneity was significant.

**Results:**

49 studies covering 4506 participants published between 2004 and 2018 were identified. Interventions included meditation, tai chi, qigong, and yoga which lasted 4 to 24 weeks. The MBTs resulted in statistically significant improvement in sleep quality and reduction on insomnia severity but no significant effects on sleep quantity indices, which were measured by sleep diary or objective measures. We analyzed the effects of tai chi and qigong separately as two different MBTs for the first time and found that qigong had a slight advantage over tai chi in the improvement of sleep quality. Subgroup analyses revealed that the effect of MBTs on sleep quality in healthy individuals was larger than clinical populations. The effect of MBTs might be influenced by the intervention duration but not the frequency.

**Conclusions:**

MBTs can be effective in treating insomnia and improving sleep quality for healthy individuals and clinical patients. More high-quality and well-controlled RCTs are needed to make a better conclusion in further study.

## 1. Introduction

As a prevalent sleep disorder, insomnia has become a public health problem, including subjective sleep complaints (e.g., poor sleep quality, inadequate sleep time), difficulties in sleep onset/maintenance, waking up too early, or nonrefreshing sleep. Insomnia is associated with significant distress or daytime impairment [1, 2]. It can occur independently or with other physical disorders and diseases (e.g., cancer, hypertension) and psychiatric disorders (e.g., anxiety, depression [2]) at a high rate of comorbidity. Sleep plays an important role in individual health. The functions of the brain, the cardiovascular system, the immune system, and the metabolic system are closely associated with sleep [3–6]. According to recent epidemiological studies, almost 25% of adults had sleep complaints, 10-15% had insomnia symptoms accompanied with daytime consequences, and 6-10% met the diagnostic criteria of insomnia disorder [7–10]. Thus, it is necessary to find effective therapies for insomnia.

Pharmacological treatment and cognitive behavioral therapy for insomnia (CBT-I) are widely used and have shown effectiveness. Pharmacotherapy is a traditional treatment for insomnia and has been tested and proven to improve sleep outcomes. Due to the risks of daytime residual effects and substance dependence, nonpharmacological treatments have attracted clinicians' attention [11, 12]. CBT-I is an effective nonpharmacological treatment that is most commonly used for insomnia. Many studies have shown that CBT-I can significantly improve sleep quality and reduce insomnia severity [13–15]. However, CBT-I is intensive, requiring administration by highly trained therapists [16]. Many other mind-body therapies (MBTs) also have effects on mitigating insomnia and produce various psychological and health functioning benefits. Examples include mindfulness meditation [17–19], tai chi [20–22], yoga [23, 24], relaxation therapy [25, 26], and music [27]. In this paper, we focus on four types of MBTs—meditation, tai chi, qigong, and yoga—which have been researched in a large number of studies and are widely used for clinical patients and community populations.

As an ancient practice, meditation is part of many spiritual traditions and types that emphasize training the mind, especially attention [28, 29]. Mindfulness meditation is mostly researched and used in both clinical and normal populations. It has also been mostly researched in mindfulness-based stress reduction (MBSR) and other variants of this practice, such as mindfulness-based cognitive therapy (MBCT) [18], mindfulness-based psychological care (MBPC) [30], mindful awareness practices (MAPs) [17, 31], and meditation awareness training (MAT) [32]. Mindfulness meditation guides individuals to pay attention to present moment experiences with openness, curiosity, and nonjudgment [29].

Tai chi, qigong, and yoga belong to meditative movements, which combined some forms of movements or body postures that focus on breathing with a clear or a calm state of mind [33]. Tai chi and qigong originated from China as martial arts based on traditional Chinese medicine [29, 34]. Both tai chi and qigong focus on incorporating the body and the mind as parts of an interconnected system and combining specific postures and movements with deep diaphragmatic breathing and mental focus to promote the mind-body interaction [29, 33–36]. Previous studies combined tai chi and qigong for analysis [33]. As two types of MBTs, tai chi and qigong differ in training methods and essentials, postures, movement characteristics, purpose, and function [37]. Therefore, different results may be produced if tai chi and qigong are analyzed separately. As one of the meditative movements, yoga has its origins in ancient India and has gained popularity among adults over the last two decades. Yoga also concentrates on the body-mind integration. In recent years, a growing number of studies have reported the abovementioned MBTs' promising results for physical and mental health, including improving sleep quality [31, 38–40] and reducing insomnia severity [19, 41–44].

In recent years, some systematic reviews have also been conducted with or without a meta-analysis of the cited issues. However, in these studies, only a small part of the evidence has been covered. They have only involved either a specific subpopulation or a certain type of therapy. Thus, it is difficult to draw broader conclusions. Furthermore, most of the existing meta-analyses have only used posttreatment scores, regardless of the existence of the baseline differences, leading to inexact results. In this study, we aim to examine the evidence that MBTs may have effects on improving the sleep health of patients with insomnia and adults who have sleep complaints and to produce an overall picture of contemporary research on this field by making a simple comparison of each intervention. We conduct this systematic review and meta-analysis of several randomized controlled trials (RCTs), which were published up to July 2018.

## 2. Methods

### 2.1. Data Sources and Study Selection

Literature searches were performed in PubMed, EMBASE, and the Cochrane Library, including studies published until July 2018. The following combinations of keywords were used: (*mind-body therapies* OR* mindfulness* OR* meditation* OR* yoga* OR* tai chi* OR* qigong*) and (*insomnia* OR* sleep disturbance* OR* sleep disorder*).

The titles and the abstracts of all publications obtained from the search strategies were screened by two reviewers. The eligibility criteria follow the PICOS framework [45].

*Participants.* The participants were adults aged 18 years or older, with active sleep disturbance documented by standard subjective measures—the Pittsburgh Sleep Quality Index (PSQI) [46] (total score>5) and the Insomnia Severity Index (ISI) [47] (total score>7)—or who were diagnosed with insomnia or had sleep disturbance that was comorbid with other diseases. People who had subjective sleep complaints without a clinical diagnosis were also included.

*Interventions.* Four approaches, including meditation, tai chi, qigong, and yoga, alone or in combination, were reviewed.

*Controls.* Both inactive (usual care or wait-list) and active (pharmacotherapy and cognitive behavioral therapy) control conditions were considered. However, one-arm studies were excluded.

*Outcomes.* Sleep-related data should be presented at both the baseline and the posttreatment, or the changed scores from the baseline to the posttreatment should be recorded, including the subjective (PSQI and sleep diary) and the objective sleep parameters (polysomnography [PSG] and actigraphy).

*Studies.* Only RCTs were included.

### 2.2. Data Extraction and Study Quality Assessment

Two reviewers independently screened the titles and the abstracts of the studies generated from the search to test whether these qualified for review. Next, the full texts were obtained and assessed according to prespecified eligibility criteria. If the reviewers had any disagreement, the third reviewer would resolve the issue by discussing it with them. The data were extracted by using data extraction forms, which were designed upfront. One reviewer (XW) extracted the data into the structured forms; the other reviewer (PL) verified their completeness and accuracy. The extracted data included the author(s); the publication year; the participant characteristics; the intervention types, frequency, duration and dropout rates; outcome measurements; and the main outcomes. We used Engauge Digitizer 10.4 to extract the data if they only showed figures in the study.

The Cochrane Risk of Bias tool [48] was used to assess the risk of bias, including selection bias (random sequence generation, allocation concealment), performance bias (blinding of participants and personnel), detection bias (blinding of outcome assessment), attrition bias (incomplete outcome data), reporting bias (selective outcome reporting), and other types of bias. Each item was assessed as high, unclear, or low risk.

### 2.3. Statistical Analysis

Stata version MP/14.2 was used for the data analysis. Because of the various baseline values of the studies' participants, we used the changed scores (from baseline to posttreatment) to calculate standardized mean differences (SMDs) and 95% confidence intervals (CIs). We used the global estimation of r = 0.5 as the correlation coefficient between posttreatment and pretreatment scores.

The magnitude of the SMDs indicated the following: (0-0.2) = negligible effect, (0.2-0.5) = small effect, (0.5-0.8) = moderate effect, and (0.8+) = large effect [49]. Heterogeneity was estimated with the I^2^ statistic. The random effect model would be used if I^2^⩾50% or the P value⩽0.1, which would indicate notable heterogeneity. Otherwise, we used the fixed effect model. Subgroup analyses were performed according to the different intervention types (meditation, tai chi, qigong, and yoga), control groups (active or inactive control conditions), and population types (clinical patients or healthy individuals).

## 3. Results

### 3.1. Search Results

**Figure 1:**
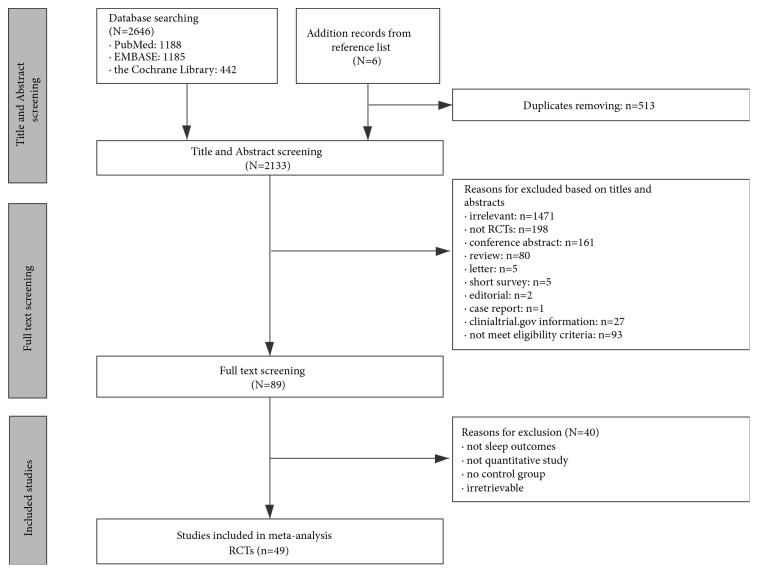
*Flowchart of trial selection process*. RCTs: randomized controlled trials.

In total, 2646 potentially relevant records were retrieved (1,188 from PubMed, 1,185 from EMBASE, and 442 from the Cochrane Library). After eliminating duplicates, the relevant records were reduced to 2,133, and 2,044 were then excluded from the review for various reasons. Of the 89 full-text articles assessed for eligibility, 43 were retained. Additionally, six articles were included from some of the selected studies' reference lists. Ultimately, 49 studies involving a total of 4506 participants were included in the meta-analysis.  Figure 1 summarizes the detailed selection process.

### 3.2. Characteristics of Included Studies

Table 1 summarizes the characteristics of the included studies. In brief, the 49 RCTs were published between 2004 and 2018. The types of participants included healthy individuals and patients, with their mean ages ranging from 35 to 78 years. The interventions included meditation (15 studies), tai chi (12 studies), qigong (4 studies), and yoga (16 studies). Two studies incorporated qigong and tai chi as the components of an integrated intervention program (QG/TC). The comparisons included no intervention, placebo, education, pharmacotherapy, CBT-I, and other exercises. The intervention duration varied from 4 weeks to 24 weeks. The sleep-related outcomes were measured by subjective measures (PSQI, ISI, and a sleep diary) and objective measures (PSG and actigraphy).

### 3.3. Risks of Bias of Included Studies

**Figure 2:**
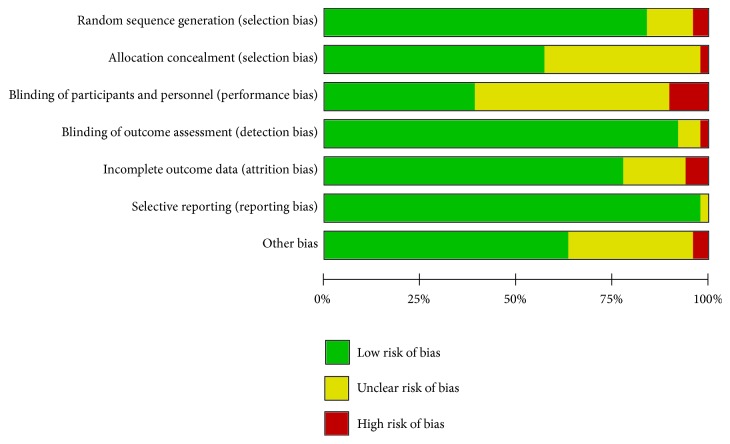
Risk of Bias Analysis.

Figure 2 presents the analysis of the risks of bias. Only 15 of the 49 studies were universally assessed as having a low risk of bias across all domains. The random sequence generation generally followed accepted methods (41 studies or 83.7%), and 28 studies (57.1%) had adequately concealed allocation. Many studies did not report whether they used blinding techniques, possibly because the authors assumed that blinding was not feasible due to the nature of the intervention. As for blinding of the outcome assessments, 45 studies (91.8%) were evaluated as low risks because the outcomes were almost assessed by self-reported scales or objective measures (PSG and actigraphy). Regarding the bias from incomplete outcome data, 38 studies (77.6%) had low risks because they reported low dropout rates or used an appropriate statistical method to account for dropouts (e.g., intention-to-treat analysis). The bias from selective reporting was assessed as low if all presetting outcomes were reported. Under this criterion, 48 studies (98.0%) were assessed as low risk on this domain.

### 3.4. Meta-Analysis Results

In this meta-analysis, the specific outcome variables included the sleep quality, the insomnia severity, which were measured by subjective measures (PSQI and ISI) and sleep quantity, such as total sleep time (TST), sleep onset latency (SOL), wake time after sleep onset (WASO), and sleep efficiency (SE), which were calculated by objective measures (PSG, actigraphy) or a sleep diary. Not all the included studies reported follow-up effects, and the follow-up period also differed. Thus, our meta-analysis aimed to evaluate the immediate postintervention effects of the four types of MBTs.

**Figure 3:**
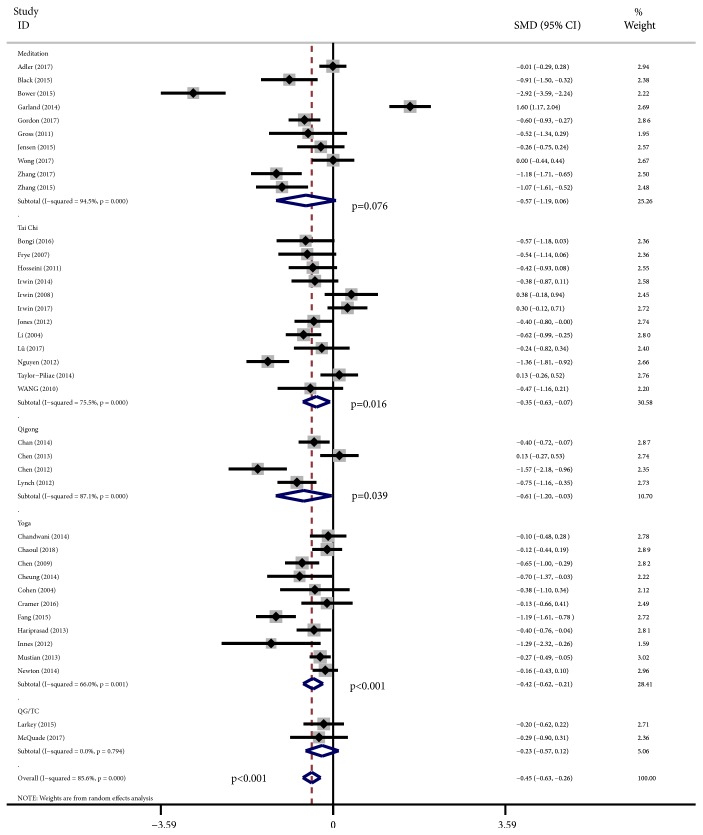
Forest plots of effect estimates of MBTs versus controls on PSQI.

**Figure 4:**
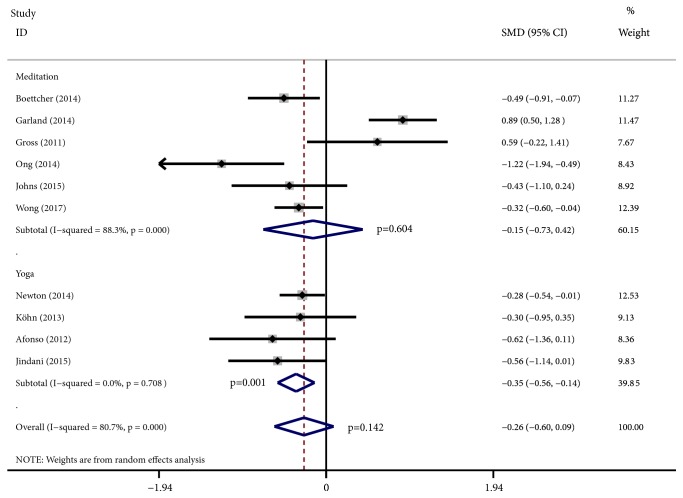
Forest plots of effect estimates of MBTs versus controls on ISI.

Figure 3 presents the overall effects of the MBTs as shown on the PSQI. Of the 49 studies, 39 studies with a total of 3,766 participants used the PSQI to assess the MBTs' effects on sleep quality. We found notable heterogeneity (I^2^⩾50%); thus, the random effects model was used. The results demonstrated the intervention group's statistically significant overall effect compared with various control conditions (effect size: -0.45; 95% CI: -0.63 to -0.26; p<0.001), with an I^2^ of 85.6%. Specifically, the results indicated that tai chi, qigong, and yoga had SMDs of -0.35 (95% CI, -0.63 to -0.07), -0.61 (95% CI, -1.20 to -0.03), and -0.42 (95% CI, -0.62 to -0.21), respectively, which were significant effects in favor of each experimental group (p = 0.016, p = 0.039, and p<0.001, respectively), but meditation had a nonsignificant effect (effect size: -0.57; 95% CI: -1.19 to 0.06; p=0.076). The mean effect sizes for the remaining sleep parameters were also nonsignificant.  Figure 4 shows the effects of MBTs' effects as shown on the ISI. Of the 49 studies, ten studies that included a total of 926 participants used the ISI, which obtained an SMDs of -0.26 (95% CI, -0.60 to 0.09), with an I^2^ of 80.7%, but the effect was nonsignificant (p = 0.142). However, the results indicated yoga's statistically significant effect (effect size: -0.35; 95% CI: -0.56 to -0.14; p = 0.001). The results of the remaining sleep parameters assessed by objective measures were -0.02 (95% CI, -0.30 to 0.25; p = 0.87) for SE, 0.05 (95% CI, -0.17 to 0.28; p = 0.48) for SOL, 0.07 (95% CI, -0.17 to 0.32; p = 0.56) for TST, and 0.11 (95% CI, -0.22 to 0.45; p = 0.50) for WASO. The results of the sleep parameters assessed by a sleep diary were 0.12 (95% CI, -0.38 to 0.63; p = 0.632) for SE, -0.02 (95% CI, -0.38 to 0.35; p = 0.934) for SOL, 0.24 (95% CI, -0.04 to 0.52; p = 0.091) for TST, and 0.49 (95% CI, -0.18 to 1.16; p = 0.150) for WASO.

These nonsignificant outcomes needed further examination since they might be influenced by the different types of control conditions. In the included trials, the control conditions differed, including alternative active treatment control and wait-list control and other inactive control conditions. For example, the forest plots of the ISI easily showed that the SMDs obtained by Garland [13] and Gross [19] were 0.89 (95% CI, 0.50 to 1.28) and 0.59 (95% CI, -0.22 to 1.41), respectively, in favor of the control interventions. In these two trials, Garland used CBT-I to compare it with meditation, while Gross employed pharmacotherapy as the control intervention, and the control conditions of these trials were both active treatments. For this reason, we needed to eliminate the influence of this factor to obtain more accurate results.

### 3.5. Subgroup Analyses

Based on the abovementioned results, we needed to conduct subgroup analyses because of the interference caused by the active control interventions. We found that when compared with the inactive control conditions, the of MBTs' efficacy in alleviating insomnia could be fully demonstrated. The results of the subgroup analyses showed many statistically significant effects on different sleep parameters, as follows: -0.36 (95% CI, -0.56 to -0.15; p=0.001) for insomnia severity measured by the ISI, -0.58 (95% CI, -0.79 to -0.36; p<0.001) for sleep quality measured by the PSQI, and -0.44 (95% CI, -0.77 to -0.11; p=0.008) for SOL measured by a sleep diary. However, there were no statistically significant differences in the pooled results of the SMDs among SE, SOL, TST, and WASO, which were calculated by objective measures (PSG and actigraphy), as well as among SE, TST, and WASO, which were assessed by means of a sleep diary.

It is worth mentioning that the efficacy of meditation, qigong, and yoga in treating insomnia was significant when compared with inactive control conditions. Meditation, qigong, and yoga had respective SMDs of -1.06 (95% CI, -1.96 to -0.17; p=0.02), -0.61 (95% CI, -1.20 to -0.03; p=0.039), and -0.39 (95% CI, -0.59 to -0.18; p<0.001) on the PSQI ranging from small to large effects. In contrast, tai chi had a nonsignificant effect (effect size: -0.55; 95% CI: -1.23 to 0.13; p = 0.091). Regarding the heterogeneity aspects, we found that I^2^<50% or even I^2^ = 0, and p>0.1 in some subgroup analyses, such as SOL (I^2^=0.0%, p=0.513) and TST (I^2^=0.0%, p=0.419), which were assessed by objective measurements, SOL (I^2^=7.0%, p=0.341), which was assessed by means of a sleep diary, and ISI (I^2^=0.0%, p=0.838). Thus, we used the fixed effect model to conduct the abovementioned subgroup analyses and used the random effect model for the remaining subgroup analyses. All the SMDs and the heterogeneity of the subgroup analyses are shown in Tables 2 and 3.

Further subgroup analyses were conducted to explore the MBTs' effects, as shown on the PSQI, among different populations. Stratified by population types, the subgroup analyses demonstrated that the studies involving clinical patients and healthy individuals both showed significant effects on sleep quality (PSQI scores), and studies involving healthy individuals had larger mean effect sizes (effect size: -0.58; 95% CI: -0.85 to -0.30; p<0.001; I^2^ = 82.6%) compared with studies involving clinical patients (effect size: -0.38; 95% CI; -0.62 to -0.14; p = 0.002; I^2^ = 86.6%). However, there was no significant difference in the pooled effect sizes between the two subgroups (P_between_ = 0.15). Other subgroup analyses based on the duration of intervention and the frequency of intervention showed no significant differences.

## 4. Discussion

To our best knowledge, this is the largest meta-analysis with the aim of examining the effects of MBTs (meditation, tai chi, qigong, and yoga) on insomnia symptoms and sleep quality among subjects with or without diseases or pre-existing conditions. The overall effects of MBTs on improving sleep quality were significant (effect size: -0.45; 95% CI: -0.63 to -0.26; p<0.001), but the effects on reducing the severity of insomnia symptoms were not significant (effect size: -0.26; 95% CI: -0.60 to 0.09; p = 0.142). These results might be influenced by the control condition type. In some studies, researchers used some active control conditions, such as CBT-I [13, 50], pharmacotherapy [19], and sleep hygiene education [31]. These active control conditions were also effective therapies or might improve sleep quality and reduce the severity of insomnia symptoms. Thus, compared with these active control conditions, MBTs might have similar effects and no obvious advantages over the former. To further explore the effects of MBTs, we conducted subgroup analyses based on the control condition type. Larger and significant SMDs could be observed when inactive control conditions were used (effect size: -0.58; 95% CI: -0.79 to -0.36; p<0.001) compared with active control conditions (effect size: -0.23; 95% CI: -0.56 to 0.10; p = 0.180) on the PSQI. As mentioned, many active control conditions were effective therapies for insomnia or could benefit sleep quality, so unsurprisingly, the effects of MBTs were not significant compared with active control conditions. Similar results were observed on the ISI. For insomnia symptoms, significant SMDs were also found when compared with inactive control (Effect size, -0.36; 95% CI, -0.56 to -0.15; p = 0.001). These results demonstrated that MBTs could be effective interventions to improve sleep quality or reduce insomnia severity and have a similar effect as those of other efficacious interventions, treatments, or exercises. However, except for SOL assessed by means of a sleep diary, the MBTs' effects on the remaining indices of sleep quantity (objectively measured SE, SOL, TST, and WASO and self-reported SE, TST, and WASO) were not significant. The reasons might be attributed to the following points: first, the various types of MBTs and their heterogeneity made it difficult to draw definite conclusions about the effectiveness of particular MBTs and might also influence the overall effects. Second, the PSQI and the ISI assessed sleep quality and the severity of insomnia symptoms, respectively. SE, SOL, TST, and WASO, which were evaluated by objective or subjective measures, were sleep quantity variables. The MBTs might contribute more to the participants' subjective feelings and experiences but might have difficulties in significantly changing the index scores. Although the results of our analysis showed that self-reported SOL was also significantly reduced, it was an individual's subjective experience as well, not objective data. Third, some included studies that assessed sleep quantity with objective measures usually had small sample sizes for various reasons, such as limited funds and patients' compliance. The small samples might lead to many difficulties in obtaining statistically significant differences between the intervention and the control conditions. We might also draw wrong conclusions, such as false positives and false negatives, because of the small samples. Finally, some improvements in TST or reductions in SOL and WASO in the control conditions were unexplained in some studies [18, 41] but might have an influence on the effects of MBTs to some extent.

We also conducted some subgroup analyses to compare the effects of MBTs based on the intervention type, the population type, and the intervention duration and frequency. For the subgroup analyses based on the population type, we compared the SMDs in the sleep quality of clinical patients and healthy people. Significant SMDs were shown in both clinical patients (effect size: -0.38; 95% CI: -0.62 to -0.14; p = 0.002) and healthy people (effect size: -0.58; 95% CI: -0.85 to -0.30; p<0.001). The effect of MBTs on the sleep quality of healthy people was obviously larger than that of clinical patients although the subgroup difference was not significant. For the clinical patients, their insomnia might be more or less related to medical disorders (e.g., knee osteoarthritis patients with chronic pain, fibromyalgia patients with non-restorative sleep, and inflammatory bowel disease [IBD] patients who must use the toilet many times/night). Thus, similar to the psychotherapies, it was difficult to solve these problems by MBTs. For the insomnia severity, MBTs had an obvious effect on reducing it among patients, but their insomnia was mostly unrelated to a medical disorder. Some examples of the treatments were MBSR or MBCT for chronic primary insomnia [19, 41, 51] and yoga for postmenopausal women with diagnosed insomnia or patients with stress-related sleep problems [43, 44]. Therefore, MBTs might be effective treatments for patients with primary or comorbid insomnia that are not caused by physical disorders, as well as for healthy people who have sleep problems. For patients whose insomnia is comorbid with physical diseases, MBTs might also be the adjuvant treatments [52]. We also believe that MBTs could be the primary preventive interventions for insomnia through stress reduction (e.g., tension, anxiety) among healthy people.

To explore the influencing factors on the effects of MBTs, we conducted subgroup analyses based on the duration and the frequency of interventions. We divided the intervention duration into *⩾*12 weeks and <12 weeks to explore the difference between them. Our results showed that the two subgroups had similar significant SMDs (effect size: -0.45; 95% CI: -0.65 to -0.25; p<0.001 for the *⩾*12-week group versus effect size: -0.45; 95% CI: -0.77 to -0.13; p=0.005 for the <12-week group). However, as mentioned, meditation-based interventions had larger SMDs (-1.06) than other MBTs, and the duration of these interventions mostly ranged from 6 to 9 weeks, which might influence our results. Therefore, we further conducted subgroup analyses among other types of MBTs (tai chi, qigong, and yoga). Our results indicated that compared with the <12-week group (effect size: -0.27; 95% CI: -0.48 to -0.07; p = 0.01), the SMDs of the *⩾*12-week group had larger effect size (effect size: -0.48; 95% CI: -0.69 to -0.27; p<0.001) although the subgroups' difference was not significant (p_between_ = 0.16). It seemed that the longer the duration of practicing MBTs was, the more positive the effect on sleep quality became. However, we could not draw this conclusion thoughtlessly, and further studies should verify the result more definitely. We also divided the intervention frequency into the *⩾*3 times/week group and the <3 times/week group to compare their SMDs. Both subgroups had significant SMDs in sleep quality, but the <3 times/week group (effect size: -0.51; 95% CI: -0.77 to -0.24; p<0.001) had a larger effect size than the *⩾*3 times/week group (effect size: -0.35; 95% CI: -0.57 to -0.13; p = 0.002). Similar to the duration, the meditation-based interventions were also mainly practiced once a week. Thus, we conducted subgroup analyses with the same method as that of the duration subgroup analyses and obtained similar results (effect size: -0.35; 95% CI: -0.57 to -0.13; p = 0.002 for the *⩾*3 times/week group versus effect size: -0.47; 95% CI: -0.69 to -0.24; p<0.001 for the <3 times/week group). These findings seemed to indicate that it would not always hold true that the higher the intervention frequency was, the better the effect became. However, the varying frequencies of the interventions in these studies made it difficult to draw a conclusion about the optimal frequency of MBTs.

We also performed subgroup analyses among the different intervention types. The two studies [53, 54] that integrated tai chi and qigong into a single intervention program had not been included in this subgroup analysis. The outcomes of specific MBTs are discussed as follows.

*Meditation*. As a prevalent mind-body exercise, meditation had become increasingly popular in recent years, which was mostly researched in MBSR and other variants of this practice. In general, these meditation practices were conducted for about 8 weeks, lasting for 2-3 hours per week. MBSR was typically taught in 2-hour weekly sessions for 8 weeks plus a full-day retreat [28]. Meditation had been proven effective in improving sleep quality and reducing the severity of insomnia. Lengacher's [55] RCT involving 79 breast cancer patients with sleep disturbances compared MBSR against the usual care and found that MBSR led to the improvement of both objective and subjective sleep parameters. Zhang et al. [30] also reported that MBSR could improve sleep quality effectively for older adults with insomnia. Other forms of meditation-based practices had also been examined and proven to be effective interventions for improving sleep quality. Britton et al. [18] performed an 8-week MBCT for 23 antidepressant medication (ADM) users with sleep complaints and found that the MBCT participants improved on both PSG and subjective measures of sleep, such as reduction in wake time and improvement in SE. Another study involving 33 leukemia patients showed that MBPC significantly improved their sleep quality [56]. Bower et al. [17] and Black et al. [31] used MAPs among younger breast cancer survivors (n = 39) and older adults with moderate sleep disturbances (n = 24), respectively. Both results showed that MAPs significantly improved the participants' sleep quality. In their RCT, Gordon et al. [32] applied MAT to improve the sleep quality of fibromyalgia patients (n = 74); undoubtedly, there was significant improvement. In sum, these meditation-based interventions could be effective in improving the sleep quality of various people. The results of our meta-analysis also support this conclusion. In our meta-analysis, we included both MBSR and other types of meditation practices. Our results demonstrated that meditation significantly improved sleep quality compared with inactive control conditions, with a large effect size of -1.06 (95% CI, -1.96 to -0.17; p = 0.02) on the PSQI, consistent with the findings of previous reviews on meditation studies for sleep improvement. Gong H. et al [57] explored the effects of mindfulness meditation on insomnia and supported the evidence about mindfulness meditation's significant effect on the improvement of sleep quality (effect size: -1.09; 95% CI: -1.50 to 0.69; p = 0.001). For the reduction of insomnia symptoms, meditation also showed a significant effect. Ong [41] reported in his study that MBSR could significantly reduce the severity of chronic insomnia. Similar results were also found in MBSR used for treating insomnia that was comorbid with cancer [13] and for persistently fatigued cancer survivors [52], as well as MBCT for chronic insomnia and Internet-Based Mindfulness Treatment for anxiety disorder [58]. Consequently, meditation could also be an effective treatment for insomnia. Some studies compared the effect of meditation with CBT-I on insomnia. An RCT showed that MBSR might produce similar clinically significant improvements; the treatment effects were not inferior to CBT-I and remained even after five months [13]. Another study demonstrated that both mindfulness-based cancer recovery (MBCR) and CBT-I produced similar levels of reduction in insomnia severity [59]. In terms of the potential mechanisms of two therapies, this study also found that the CBT-I program, similar to MBCR, also improved mindfulness unexpectedly. The authors also reported that the insomnia severity of the MBCR participants continued to lessen over time, while the CBT-I participants might have experienced a weakening of the treatment effect over the follow-up period. As a result, meditation could be treated as an effective alternative method to improve sleep quality and treat insomnia.

*Tai Chi*. As a form of mind-body exercise, tai chi has become popular over the last three decades, with its calm, low-impact, and integrated movements. Tai chi includes many types and is typically conducted about one to three times a week (1-2 hours per session) for 12 weeks or longer. Owing to the differences in the forms of tai chi, its duration and frequency also varied. Tai chi had been proven effective in improving self-reported sleep and reducing insomnia severity in adult and elderly populations [33] and was mostly aimed at older people. Nguyen and Kruse [60] concluded in their RCT covering 96 subjects that tai chi was an effective nonpharmacological treatment to enhance the sleep of elderly Vietnamese with sleep disturbances. Irwin [61] implemented a twice-weekly Tai Chi Chih program (TCC) for 16 weeks and found that compared with the sleep seminar education control (SS) TCC produced improvements in the global sleep quality of the elderly. Sarris and Byrne's review supported the evidence that tai chi improved sleep quality [62]. Similar effects had also been found in middle-aged populations [63], but the applications of tai chi among the young generation were fewer. In their meta-analysis, Irwin, Cole, and Nicassio [64] reported that tai chi intervention had a better effect (effect size = 0.89; 95% CI: 0.28 to 1.50) on sleep quality than other regular exercises, which might be attributed to its mind-body form [34]. Our included studies also support this result. An RCT from Li et al. [65] compared tai chi with low-impact exercise and found that tai chi participants reported significant improvements in five of the PSQI subscale scores (sleep quality, sleep onset latency, sleep duration, sleep efficiency, sleep disturbances) (P<0.01) and PSQI global score (P=0.001). Irwin et al. [50] also compared the Tai Chi Chih program (TCC) with CBT-I and evaluated them in months 2, 3 (posttreatment), 6, and 15 (follow-up). Their study showed that the TCC was not inferior to CBT-I at 15 (P = 0.02), 3 (P = 0.02), and 6 (P<0.01) months. The insomnia remission rates in CBT-I and the TCC were 46.2% and 37.9%, respectively. Thus, Irwin et al. concluded that tai chi was statistically not inferior to CBT-I and produced clinically meaningful improvements in reducing insomnia. In our meta-analysis, the effect of tai chi on the improvement of sleep quality compared with all control groups had the SMDs of -0.35 (95% CI: -0.63 to -0.07; p=0.016) on the PSQI. This result further proved that tai chi could produce significant effects on improving sleep quality despite the overall small effect size. It could be treated as a more effective intervention compared with other regular exercises.

*Qigong*. Qigong includes various types. Translated from Chinese, “qi” means energy flow, which is considered as the inherent functional and energetic essence of human beings in traditional Chinese medicine, and “gong” means skills or achievements; roughly, qigong means “to cultivate qi” [66, 67]. Relative to other MBTs, qigong has been less studied in relation to insomnia, but it has been proven effective. Among the 49 included studies, 6 applied qigong. Lynch's [68] study involving 100 fibromyalgia patients demonstrated that* Chaoyi Fanhuan Qigong* (CFQ) significantly reduced the total PSQI global score. Another RCT involving 72 perimenopausal women with sleep disturbances showed that* Ping Shuai Qigong* resulted in the improvement of sleep quality and climacteric symptoms at 6 weeks and 12 weeks [38]. Chen et al. [69] performed an intervention using* Baduanjin Qigong* for 56 older people and found that the* Baduanjin* exercise group reported significantly better sleep quality after 4 weeks of intervention, which was maintained throughout the 12-week exercise period. Chan et al. [70] also proved that* Baduanjin* qigong was an efficacious and acceptable treatment for sleep disturbance in Chronic Fatigue Syndrome-Like Illness. Although qigong was proven effective in these studies, other studies reported nonsignificant differences in the sleep quality of breast cancer survivors [53, 71]. Based on our meta-analysis, 5-12 weeks (1-7 times/week, totaling 30-120 minutes weekly) of qigong demonstrated a moderate effect and a statistically significant decrease in the PSQI score compared with all control groups (effect size = -0.61; 95% CI, -1.20 to -0.03; p = 0.039; I^2^ = 87.1%). All of these studies used inactive control conditions. Previous reviews also combined qigong with tai chi for analysis. Wu et al. [33] showed that tai chi/qigong had a moderate effect on the improvement of sleep quality (effect size = -0.64; 95% CI: -0.97 to -0.30; p<0.01). In our included studies, two incorporated tai chi and qigong as an integrated intervention program (QG/TC) [53, 54]. However, as mentioned, tai chi and qigong had numerous differences, so they might produce various effects on sleep quality or insomnia; thus, it was necessary to analyze them separately. According to our meta-analysis, qigong showed significant effects on improving sleep quality, indicating that it could be treated as an effective intervention for improving sleep quality.

*Yoga. *In recent years, a growing number of scientific investigations have shown that practicing yoga could produce potential benefits for healthy and clinical populations [72] and improve sleep quality [42, 73] and reduce insomnia symptoms [43, 74]. In their study involving 410 cancer survivors with moderate to high sleep disturbances, Mustian KM et al. [75] showed that an eight-session yoga program improved the participants' sleep outcomes. Newton [42] also demonstrated that a 12-week yoga class plus home practice reduced insomnia symptoms compared with the usual activity set for women with menopausal vasomotor symptoms. Yoga also brought benefits for elderly people. Chen et al. [76] showed that yoga significantly improved the sleep quality of older adults with sleep complaints. An RCT [77] involving older knee osteoarthritis patients demonstrated that weekly yoga mitigated their sleep disturbances, but their PSQI score declined significantly at 20 weeks. In a recent meta-analysis [33], yoga was proven to have a statistically significant moderate effect on the sleep quality of the elderly (effect size = -0.77; 95% CI: -1.08 to -0.46; p<0.01). Each of these cited studies only targeted a specific population. However, in our meta-analysis, the studies had various population types, and we found a small effect and a statistically significant reduction in the PSQI score (effect size = -0.42; 95% CI: -0.62 to -0.21; p<0.001) and the ISI score (effect size = -0.35; 95% CI: -0.56 to -0.14; p = 0.001) compared with all control groups. Nevertheless, yoga was still treated as an effective treatment for reducing insomnia symptoms and improving sleep quality.

Much evidence demonstrated that MBTs might produce benefits for different groups of people, such as insomnia patients [19, 41], cancer survivors [17, 50, 53, 71, 75, 78], fibromyalgia patients [22, 32, 63, 68], depressed patients [18, 79], postmenopausal women [43], and older adults [20, 24, 30, 31, 39, 60, 65, 69, 76]. Moreover, previous systematic or narrative reviews had shown that many types of MBTs could improve sleep quality and reduce insomnia severity [62, 80]. The results of our meta-analysis were roughly in line with these reviews' findings. The meta-analysis of Gong H. et al. [57] demonstrated that mindfulness meditation significantly improved sleep quality, with the SMD of -1.09 (95% CI, -1.50 to 0.69; p<0.001). Raman and Zhang [34] showed that tai chi also had a large effect on and a statistically significant improvement in the sleep quality of healthy adults and patients with chronic conditions (effect size = 0.89; 95% CI: 0.28 to 1.50). Wu et al. [33] found that meditative movement intervention (MMI) produced a moderate effect on the elderly (effect size = -0.70; 95% CI: -0.96 to -0.43; p<0.001). Furthermore, the effect size of MBTs on improving self-reported sleep quality was similar to those of other treatment modalities. For example, a systematic review indicated that exercise could enhance the sleep quality of middle-aged and older adults with sleep problems (effect size = 0.47; 95% CI: 0.08 to 0.86) [81]. Irwin's [64] review revealed that behavioral intervention significantly improved sleep quality (effect size = 0.60; 95% CI, 0.19 to 1.01), which was similar to the finding from our meta-analysis. For the specific MBTs, we found that meditation had a larger effect than tai chi, qigong, and yoga. It should be mentioned that we analyzed tai chi and qigong separately for the first time. Our results showed qigong as a proven effective intervention for improving sleep quality, which had a larger SMD than tai chi (effect size = -0.61; 95% CI: -1.20 to -0.03 versus effect size = -0.35; 95% CI: -0.63 to -0.07). These results seemed to further corroborate our previous hypothesis that tai chi and qigong might produce different effects on the improvement of sleep quality even though they both came from traditional Chinese medicine. From our subgroup analyses, we further found that qigong (effect size: -0.61; 95% CI: -1.20 to -0.03; p = 0.039) had a slight advantage over tai chi (effect size: -0.55; 95% CI: -1.23 to 0.13; p = 0.116) compared with inactive control conditions, but tai chi's effect was not significant. Nevertheless, as two different MBTs, tai chi and qigong should not be equated. Their comparative analysis should be further explored to draw a more explicit conclusion.

According to our additional subgroup analyses, the effect of MBTs on the sleep quality of healthy adults was larger compared with clinical patients. This result might be influenced by the patients' characteristics. For those patients whose insomnia is caused by other medical disorders, MBTs may not achieve the desired effect. Treating their related medical disorder is the fundamental way to reduce their insomnia. Therefore, for these patients, MBTs might only be used as adjuvant therapies. In sum, MBTs could be treated as effective preventive interventions for insomnia in both healthy and clinical populations. MBTs could also be used as adjuvant or alternative therapies in treating insomnia with or without comorbidity, respectively. However, because secondary insomnia is always associated with physical or mental disorders, which is not the case of primary insomnia, this difference might interfere with the outcomes. Further studies should separate primary insomnia from secondary insomnia to explore the MBTs' effect on insomnia in clinical populations. Our other subgroup analyses showed that the effects of MBTs might be influenced by the intervention duration but not the frequency, and these results should be confirmed in the future research.

Mild to moderate dropout rates were also founded in these studies. According to the included studies, the dropout rates greatly varied; 6 studies [13, 41, 43, 65, 74, 82] had high dropout rates (*⩾*30%), 2 [63, 83] had not reported any dropout rate, and others had low to moderate dropout rates. We also calculated that the mean dropout rates were 14.03% in meditation (15 studies), 8.47% in qigong (4 studies), 14.33% in tai chi (11 studies, 1 study did not report any), 15.72% in QG/TC (2 studies), and 16.93% in yoga (15 studies, 1 study did not report any).

## 5. Advantages and Limitations

Our study had several strengths. First, we included 49 studies in this meta-analysis, which produced more comprehensive and broader conclusions. This review included both healthy and clinical populations, ranging from young and middle-aged to older people. Second, both subjective and objective outcomes were analyzed. We extracted outcomes from a sleep questionnaire, a sleep diary, PSG, and actigraphy to conduct an overall meta-analysis, which covered both sleep quality and sleep quantity. Third, we analyzed the effects of tai chi and qigong separately, leading to more explicit results, and we further clarified the effects of each intervention on sleep quality and insomnia.

Although the findings of this meta-analysis suggested some promising clinical benefits of MBTs for alleviating insomnia, there were also several limitations. First, we only included studies published in English, which might have influenced our results to some extent and limited the generalizability of our findings. For example, the studies on the intervention of qigong were mostly included in Chinese databases; thus, the evidence on the effect of qigong on insomnia was inadequate. Second, our subgroup analysis might not have been sufficiently robust to obtain the actual effect because of the limited studies and the relatively small sample size. Third, the studies included in this meta-analysis had significant heterogeneity. The study quality, various population types, the intervention duration and frequency, and even the severity of insomnia or sleep complaints might influence heterogeneity. Finally, we only used the immediate posttreatment outcomes to examine the effects of the four types of MBTs on insomnia, but some studies showed improvements in sleep quality in the follow-up period.

## 6. Conclusions

In conclusion, this systematic review and meta-analysis provided evidence that MBTs could be effective in treating insomnia and improving the sleep quality of healthy subjects and clinical patients. As two different types of MBTs, tai chi and qigong were analyzed separately and produced a minor difference in outcomes. These results might indicate that tai chi and qigong, as two different types of MBTs, should not be equated. Our findings on the larger effect of MBTs on the sleep quality of healthy adults compared with clinical patients should also be further explored. However, we only included studies published in English, which also had varying levels of quality. Further research should include high-quality and well-controlled RCTs, published in English and other languages. Future studies should conduct more detailed subgroup analyses to confirm the accuracy of the effect sizes of MBTs; the changes observed in the follow-up period should also be considered.

## Figures and Tables

**Table 1:** Characteristics of included studies.

Study (year)	Participants	N, age	Intervention type Duration, frequency	Control condition	Sleep related outcome measures	Dropouts (%)	Study type
*Part 1 Studies used active control conditions*							
Gross 2011	Chronic primary insomnia	30, 21-65	*Meditation*-MBSR	PCT	PSQI, ISI, Actigraphy, sleep diary	10%	RCT
			2.5hr/wk, 8wk				
Boettcher 2014	Anxiety disorder	91, 38(10.3)	*Meditation*	Online discussion forum	ISI	11%	RCT
			1 module/wk, 8wk				
Garland 2014	Insomnia comorbid with cancer	111, 58.89(11.08)	*Meditation*-MBSR	CBT-I	PSQI, ISI, Actigraphy, sleep diary	50%	RCT
			90min/wk, 8wk				
Ong 2014	Chronic insomnia	35, 42.9(12.2)	*Meditation*-MBSR&MBTI	Self-monitoring condition	ISI, PSAS, PSG, Actigraphy, sleep diary	31.58%	RCT
			2.5hr/wk&6hr retreat between 5-7wk, 8wk				
Black 2015	Older adults	49, 66.34(7.4)	*Meditation*-MAPs	SHE program	PSQI; Athens Insomnia Scale	12.5%	RCT
			2hr/wk, 6wk				
Alder 2017	Adults with obesity	194, 47(12.49)	*Meditation*-MBWLI	PMR	PSQI	20%	RCT
			2-2.5hr ×16, 5.5 mons				
Gordon 2017	Fibromyalgia	148, 46.88(9.43)	*Meditation*-MAT	CBTG	PSQI	27%	RCT
			2hr/wk, 8wk				
Wong 2017	Adults with chronic primary insomnia	216, 56.09(9.4)	*Meditation*-MBCT	PEEC	ISI; sleep diary	9%	RCT
			2.5hr/wk, 8wk				
Larkey 2015	Breast cancer survivors	101, 58.8(8.94)	*Qigong/Tai Chi*-QG/TCE	Sham Qigong	PSQI	12.24%	RCT
			60min/wk, 12wk				
Li 2004	Older adults with sleep complaints	118, 75.37(7.77)	*Tai Chi*-Easy Tai Chi	Low-impact exercise	PSQI, ESS	32.26%	RCT
			60min×3/wk, 24wk				
Irwin 2008	Older adults	52, 70.12(6.68)	*Tai Chi*-TCC	Health education	PSQI	11.86%	RCT
			40min×3/wk, 16wk				
WANG 2010	Elderly with cerebrovascular disorder	34, 77.06(10.95)	*Tai Chi*-Simplified Yang-style	Rehabilitation program	PSQI	5.88%	RCT
			50min/wk, 12wk				
Jones 2012	Fibromyalgia	101, 54.04	*Tai Chi*-Modified Yang-style	Education	PSQI	0%	RCT
			1.5 hr×2/wk, 12wk				
Irwin 2014	Older adults with chronic and primary insomnia	73, 66.33(7.45)	*Tai Chi*	Sleep seminar education control	PSQI, Athens Insomnia Scale, ESS, PSG, sleep diary	16.67%	RCT
			2hr/wk, 4months				
Bongi 2016	Fibromyalgia	44, 52.24(12.19)	*Tai Chi*-Tai Ji Quan 60min×2/wk, 16wk	Educational course about FMS	PSQI	NR	RCT
Irwin 2017	Breast cancer survivors	90, 59.8(8.58)	*Tai Chi*-Tai Chi Chih	CBT-I	PSQI, AISI, ESS, PSG, sleep diary	15.56%	RCT
			2hr×8wk+1month skill consolidation				
Lü 2017	Knee osteoarthritis women	46, 64.57(3.38)	*Tai Chi*-Tai Ji Quan 60min×3/wk, 24wk	Wellness education classes	PSQI, sleep latency, total sleep time, sleep efficiency	8.70%	RCT
Innes 2012	Older Women with Restless Legs Syndrome	20, 58.7(8.1)	*Yoga*-lyengar yoga program 90min×2/wk, 8wk	Education film intervention	PSQI	20%	RCT

*Part 2 Studies used inactive control conditions*							
Britton 2012	Antidepressant users with sleep complaints	26, 46.97(7.8)	*Meditation*-MBCT	Wait-list control condition	PSG, sleep diary	6.67%	RCT
			3hr/wk, 8wk				
Johns 2015	fatigued Cancer survivors	35, 57.29(9.3)	*Meditation*-MBSR-CRF	Wait-list control condition	ISI	0%	RCT
			2hr/wk, 7wk				
Bower 2015	Younger Breast Cancer survivors	71, <50years	*Meditation*-MAPs	Wait-list control condition	PSQI	10.26%	RCT
			2hr/wk, 6wk				
Lengacher 2015	Breast cancer	79, 57(9.7)	*Meditation*-MBSR	Usual care	PSQI, Actigraphy, sleep diary	0%	RCT
			2hr/wk, 6wk				
Jensen 2015	Stressed person with poor sleep quality	72, 42(9)	*Meditation*-Open and Calm	Usual treatment	PSQI	6%	RCT
			2.5hr/wk, 9wk				
Zhang 2015	Older adults with chronic insomnia	60, 78.1(2.99)	*Meditation*-MBSR	Wait-list control condition	PSQI	3.33%	RCT
			2hr/wk, 8wk				
Zhang 2017	Leukemnia patients in chemotherapy	76, 39.03(9.14)	*Meditation*-MBPC	Conventional care	PSQI	13.16%	RCT
			30-40min/wk, 5wk				
Chen 2012	Older people	56, 71.75(8.13)	*Qigong*-Baduanjin Qigong	No treatment	PSQI	3.57%	RCT
			30min×3/wk, 12wk				
Lynch 2012	Fibromyalgia	100, 52.49(8.71)	*Qigong*-CFQ	Wait-list control condition	PSQI	16.98%	RCT
			40-60min/day, 8wk				
Chen 2013	Breast cancer	96, 45(8.1)	*Qigong*-Guo-lin New Qigong	Wait-list control condition	PSQI	0%	RCT
			40min×5/wk, 5 or 6wk				
Chan 2014	CFS patients	150, 39(7.93)	*Qigong*-Baduanjin Qigong	Wait-list control condition	PSQI	13.33%	RCT
			1.5hr×16sessions arranged over 9wk				
McQuade 2017	Prostate cancer patients undergoing radiotherapy	50, 64.23(8.1)	*Qigong/Tai Chi*-QGTC	Wait-list control condition	PSQI	19.2%	RCT
			40min×4/wk, during radiotherapy				
Frye 2007	Older adults	54, 69.2(9.26)	*Tai Chi-*Yang-style tai chi	Non-exercise control	PSQI	25.8%	RCT
			60min×3/wk, 12wk				
Hosseini 2011	Older adult residents in nursing home	62, 69.08(5.38)	*Tai Chi*-Yang-style tai chi	Routine daily activity	PSQI	12.90%	RCT
			20-25min×3/wk, 12wk				
Nguyen 2012	Older adults	96, 68.9(5.1)	*Tai Chi*	Routine daily activity	PSQI	18.75%	RCT
			1hr×2/wk, 24wk				
Taylor-Piliae 2014	Community-dwelling survivors of stroke	101, 69.9(10)	*Tai Chi*-Yang-style tai chi	Usual care	PSQI	9.43%	RCT
			60min×3/wk, 12wk				
Cohen 2004	Lymphoma patients	38, 51	*Yoga*-Tibetan yoga	Wait-list control condition	PSQI	NR	RCT
			Yoga session×1/wk, 7wk				
Manjunath 2005	geriatric population with self-reported sleep difficulty	46, 71.2(7.85)	*Yoga*	Wait-list control condition	Sleep rating questionnaire	21.74%	RCT
			60min×6/wk, 24wk				
Chen 2009	Older adults with sleep complaints	139, 68.98(6.18)	*Yoga*-sliver yoga program	Wait-list control condition	PSQI	7.46%	RCT
			70min×3/wk, 24wk				
Afonso 2012	Postmenopausal women with insomnia diagnosed	40, 50-65years	*Yoga*	Wait-list control condition	ISI	37.50%	RCT
			1hr×2/wk, 4months				
Hariprasad 2013	Elderly with sleep disturbances	120, 75.28(6.89)	*Yoga*-yoga program	Wait-list control condition	PSQI	29.03%	RCT
			60min×7/wk, 24wk				
Köhn 2013	Patients with stress-related symptoms or diagnoses	39, 53.03(12.17)	*Yoga*-medical yoga	Standard care	ISI	10%	RCT
			60min/wk, 12wk				
Mustian 2013	Cancer survivors	410, 54.1(10.33)	*Yoga*-YOCAS®	Standard care	PSQI, Actigraphy	18.45%	RCT
			75min×2/wk, 4wk				
Chandwani 2014	Breast Cancer	107, 52.24(9.79)	*Yoga*	Usual care	PSQI	7.50%	RCT
			60min×3/wk, 6wk				
Cheung 2014	Older women with knee osteoarthritis	36, 71.9	*Yoga*-Hatha yoga	Wait-list control condition	PSQI	0%	RCT
			60min/wk, 8wk				
Newton 2014	Women with menopausal vasomotor symptoms	249, 54.24(3.67)	*Yoga*	Usual activity	PSQI, ISI	1.87%	RCT
			90min/wk, 12wk				
Fang 2015	Nurse with poor sleep in China	120, 35.58(10.43)	*Yoga*	Non-yoga control group	PSQI	11.48%	RCT
			50-60min×2/wk, 6months				
Jindani 2015	Adults with Posttraumatic Stress	80, 41(18-64)	*Yoga*-Kundalini yoga	Wait-list control condition	ISI	30%	RCT
			90min/wk, 8wk				
Cramer 2016	Colorectal cancer patients	54, 68.3(9.7)	*Yoga*-Hatha yoga	Wait-list control condition	PSQI	22.22%	RCT
			90min/wk, 10wk				
Buchanan 2017	Menopausal Women with Hot Flashes	132, 54.63(3.8)	*Yoga*-based on Viniyoga	Usual activity	Actigraphy	40.38%	RCT
			90min/wk, 12wk				
Chaoul 2018	Breast cancer undergoing chemotherapy	159, 49.23(9.93)	*Yoga*-Tibetan yoga	Usual care	PSQI	13.5%	RCT
			75-90minutes×4/wk, 12wk				

*Abbreviations. PSQI*: Pittsburgh Sleep Quality Index; *ISI*: Insomnia Severity Index; *PSAS*: Presleep Arousal Scale; *AISI*: Athens Insomnia Severity Index; *ESS*: Epworth Sleepiness Scale; *SWS*: slow-wave sleep; *PSG*: polysomnography; *MBSR*: mindfulness-based stress reduction; *MBCT*: mindfulness-based cognitive therapy; *MAPs*: mindful awareness practices; *MBTI*: mindfulness-based therapy for insomnia; *MBWLI*: mindfulness-based weight loss intervention; *MAT*: meditation awareness training; *MBPC*: mindfulness-based psychological care; *PMR*: progressive muscle relaxation; *PCT*: pharmacotherapy; *CBT-I*: cognitive behavioral therapy for insomnia; *CBTG*: Cognitive behavioral theory for groups; *SHE*: sleep hygiene education; *PEEC*: psychoeducation with stretching exercise control; *CFQ*: Chaoyi Fanhuan Qigong; *RCT*: randomized controlled trials; *NR*: not report

**Table 2:** Comparison of outcome measures between MBTs and inactive control conditions.

Sleep parameters	Studies	SMDs (95% CI)	p-value	I^2^	p-value
	(n)		(overall effect)	Value (%)	(heterogeneity)
PSQI	24	-0.58 (-0.79, -0.36)	**<0.001**	85.6%	<0.001
Meditation	5	-1.06 (-1.96, -0.17)	**0.020**	93.1%	<0.001
Tai Chi	4	-0.55 (-1.23, 0.13)	0.116	87.7%	<0.001
Qigong	4	-0.61 (-1.20, -0.03)	**0.039**	87.1%	<0.001
Yoga	10	-0.39 (-0.59, -0.18)	**<0.001**	65.6%	0.002
ISI	5	-0.36 (-0.56, -0.15)	**0.001**	0.00%	0.838
Objective-SE	3	0.20 (-0.13, 0.52)	0.232	51.4%	0.041
Objective-SOL	4	-0.03 (-0.20, 0.14)	0.728	0.0%	0.513
Objective-TST	3	0.19 (-0.07, 0.45)	0.156	0.0%	0.419
Objective-WASO	4	0.07 (-0.50, 0.63)	0.816	87.3%	<0.001
Self-reported-SE	1	0.67 (-0.18, 1.52)	0.123	—	—
Self-reported-SOL	3	-0.44 (-0.77, -0.11)	**0.008**	7.0%	0.341
Self-reported-TST	3	0.49 (-0.11, 1.09)	0.106	64.8%	0.058
Self-reported-WASO	1	-0.47 (-1.31,0.37)	0.270	—	—

*Note.* Bold data indicate significant effect size.

**Table 3:** Exploratory of subgroup differences in SMDs in PSQI among included studies.

Subgroups	Studies	SMDs (95% CI)	p-value	I^2^	p-value	p-value
	(n)		(overall effect)	Value (%)	(heterogeneity)	(group difference) ^a^
Type of intervention						
Meditation	10	-0.57 (-1.19, 0.06)	0.076	94.5%	<0.001	0.830
Tai Chi	12	-0.35 (-0.63, -0.07)	**0.016**	75.5%	<0.001	
Qigong	4	-0.61 (-1.20, -0.03)	**0.039**	87.1%	<0.001	
Yoga	11	-0.42 (-0.62, -0.21)	**<0.001**	66.0%	0.001	
Type of control						
Active control	15	-0.23 (-0.56, 0.10)	0.180	86.3%	<0.001	0.080
Inactive control	24	-0.58 (-0.79, -0.36)	**<0.001**	84.3%	<0.001	
Type of participant						
Clinical patient	27	-0.38 (-0.62, -0.14)	**0.002**	86.6%	<0.001	0.210
Healthy adult	16	-0.58 (-0.85, -0.30)	**<0.001**	82.6%	<0.001	
Duration of intervention						
*⩾*12 weeks	19	-0.45 (-0.65, -0.25)	**<0.001**	77.3%	<0.001	1.000
<12 weeks	20	-0.45 (-0.77, -0.13)	**0.005**	89.7%	<0.001	
Frequency of intervention						
*⩾*3 times/week	14	-0.35 (-0.57, -0.13)	**0.002**	71.8%	<0.001	0.370
<3 times/week	25	-0.51 (-0.77, -0.24)	**<0.001**	89.0%	<0.001	

*Notes*: Bold data indicate significant effect size. ^a^ means significance of differences among subgroups

*Abbreviations*: SMDs, Standardized mean differences; CI, Confidence Interval

## Additional Points

*Highlight. (1) MBTs could be effective in improving sleep quality and treating insomnia of healthy subjects and clinical patients. (2) Meditation had a larger effect than tai chi, qigong, and yoga. As different types of MBTs, qigong had a slight advantage over tai chi on the improvement of sleep quality. Therefore, they should be analyzed separately in the future. (3) Our result of subgroup analysis performed by population types revealed that the effect of MBTs on sleep quality in healthy individuals was larger than clinical populations. (4) The effect of MBTs might be influenced by intervention duration, but a similar result was not observed in intervention frequency.*

## References

[1] Araújo T., Jarrin D. C., Leanza Y., Vallières A., Morin C. M. (2017). Qualitative studies of insomnia: Current state of knowledge in the field. *Sleep Medicine Reviews*.

[2] Morin C. M., Benca R. (2012). Chronic insomnia. *The Lancet*.

[3] Hanlon E. C., Van Cauter E. (2011). Quantification of sleep behavior and of its impact on the cross-talk between the brain and peripheral metabolism. *Proceedings of the National Acadamy of Sciences of the United States of America*.

[4] Bryant P. A., Trinder J., Curtis N. (2004). Sick and tired: Does sleep have a vital role in the immune system?. *Nature Reviews Immunology*.

[5] Xie L., Kang H., Xu Q. (2013). Sleep drives metabolite clearance from the adult brain. *Science*.

[6] Dolgin E. (2013). Deprivation: A wake-up call. *Nature*.

[7] Morin C. M., LeBlanc M., Daley M., Gregoire J. P., Mérette C. (2006). Epidemiology of insomnia: prevalence, self-help treatments, consultations, and determinants of help-seeking behaviors. *Sleep Medicine*.

[8] Ohayon M. M. (2002). Epidemiology of insomnia: what we know and what we still need to learn. *Sleep Medicine Reviews*.

[9] Roth T., Jaeger S., Jin R., Kalsekar A., Stang P. E., Kessler R. C. (2006). Sleep problems, comorbid mental disorders, and role functioning in the national comorbidity survey replication. *Biological Psychiatry*.

[10] Ohayon M. M., Reynolds C. F. (2009). Epidemiological and clinical relevance of insomnia diagnosis algorithms according to the DSM-IV and the International Classification of Sleep Disorders (ICSD). *Sleep Medicine*.

[11] Kamel N. S., Gammack J. K. (2006). Insomnia in the elderly: Cause, approach, and treatment. *American Journal of Medicine*.

[12] Montgomery P., Dennis J. (2004). A systematic review of non-pharmacological therapies for sleep problems in later life. *Sleep Medicine Reviews*.

[13] Garland S. N., Carlson L. E., Stephens A. J., Antle M. C., Samuels C., Campbell T. S. (2014). Mindfulness-based stress reduction compared with cognitive behavioral therapy for the treatment of insomnia comorbid with cancer: A randomized, partially blinded, noninferiority trial. *Journal of Clinical Oncology*.

[14] Morin C. M., Culbert J. P., Schwartz S. M. (1994). Nonpharmacological interventions for insomnia: a meta-analysis of treatment efficacy. *The American Journal of Psychiatry*.

[15] Black D. S., O'Reilly G. A., Olmstead R., Breen E. C., Irwin M. R. (2014). Mindfulness-based intervention for prodromal sleep disturbances in older adults: Design and methodology of a randomized controlled trial. *Contemporary Clinical Trials*.

[16] Morin C. M., Bootzin R. R., Buysse D. J., Edinger J. D., Espie C. A., Lichstein K. L. (2006). Psychological and behavioral treatment of insomnia: update of the recent evidence (1998–2004). *SLEEP*.

[17] Bower J. E., Crosswell A. D., Stanton A. L. (2014). Mindfulness meditation for younger breast cancer survivors: a randomized controlled trial. *Cancer*.

[18] Britton W. B., Haynes P. L., Fridel K. W., Bootzin R. R. (2012). Mindfulness-based cognitive therapy improves polysomnographic and subjective sleep profiles in antidepressant users with sleep complaints. *Psychotherapy & Psychosomatics*.

[19] Gross C. R., Kreitzer M. J., Reilly-Spong M. (2011). Mindfulness-based stress reduction versus pharmacotherapy for chronic primary insomnia: a randomized controlled clinical trial. *Explore: The Journal of Science and Healing*.

[20] Irwin M. R., Olmstead R., Motivala S. J. (2008). Improving sleep quality in older adults with moderate sleep complaints: a randomized controlled trial of Tai Chi Chih. *SLEEP*.

[21] Wang W., Sawada M., Noriyama Y. (2010). Tai Chi exercise versus rehabilitation for the elderly with cerebral vascular disorder: a single-blinded randomized controlled trial. *Psychogeriatrics*.

[22] Jones K. D., Sherman C. A., Mist S. D., Carson J. W., Bennett R. M., Li F. (2012). A randomized controlled trial of 8-form Tai chi improves symptoms and functional mobility in fibromyalgia patients. *Clinical Rheumatology*.

[23] Manjunath N. K., Telles S. (2005). Influence of Yoga & Ayurveda on self-rated sleep in a geriatric population. *Indian Journal of Medical Research*.

[24] Halpern J., Cohen M., Kennedy G., Reece J., Cahan C., Baharav A. (2014). Yoga for improving sleep quality and quality of life for older adults. *Alternative Therapies in Health and Medicine*.

[25] Lichstein K. L., Riedel B. W., Wilson N. M., Lester K. W., Aguillard R. N. (2001). Relaxation and sleep compression for late-life insomnia: A placebo-controlled trial. *Journal of Consulting and Clinical Psychology*.

[26] Waters W. F., Hurry M. J., Binks P. G. (2003). Behavioral and hypnotic treatments for insomnia subtypes. * Behavioral Sleep Medicine*.

[27] Harmat L., Takács J., Bódizs R. (2008). Music improves sleep quality in students. *Journal of Advanced Nursing*.

[28] Chaoul A., Milbury K., Sood A. K., Prinsloo S., Cohen L. (2014). Mind-body practices in cancer care. *Current Oncology Reports*.

[29] Bower J. E., Irwin M. R. (2016). Mind-body therapies and control of inflammatory biology: a descriptive review. *Brain, Behavior, and Immunity*.

[30] Zhang J., Liu X., Xie X. (2015). Mindfulness-based stress reduction for chronic insomnia in adults older than 75 years: A randomized, controlled, single-blind clinical trial. *Explore: The Journal of Science and Healing*.

[31] Black D. S., O'Reilly G. A., Olmstead R., Breen E. C., Irwin M. R. (2015). Mindfulness meditation and improvement in sleep quality and daytime impairment among older adults with sleep disturbances: A randomized clinical trial. *JAMA Internal Medicine*.

[32] Van Gordon W., Shonin E., Dunn T. J., Garcia-Campayo J., Griffiths M. D. (2016). Meditation awareness training for the treatment of fibromyalgia syndrome: A randomized controlled trial. *British Journal of Health Psychology*.

[33] Wu W.-W., Kwong E., Lan X.-Y., Jiang X.-Y. (2015). The effect of a meditative movement intervention on quality of sleep in the elderly: A systematic review and meta-analysis. *The Journal of Alternative and Complementary Medicine*.

[34] Raman G., Zhang Y., Minichiello V. J. (2013). Tai Chi improves sleep quality in healthy adults and patients with chronic conditions: A systematic review and meta-analysis. *Journal of Sleep Disorders & Therapy*.

[35] Wang C. (2011). Tai Chi and rheumatic diseases. *Rheumatic Disease Clinics of North America*.

[36] Yang Y., Li X. Y., Gong L., Zhu Y. L., Hao Y. L. (2014). Tai Chi for improvement of motor function, balance and gait in Parkinson’s disease. *PLOS ONE*.

[37] Li Z., Xu M. (2013). Analysis on the difference between Tai Chi and health qigong. *Contemporary Sports Technology*.

[38] Yeh S.-C. J., Chang M.-Y. (2012). The effect of qigong on menopausal symptoms and quality of sleep for perimenopausal women: A preliminary observational study. *The Journal of Alternative and Complementary Medicine*.

[39] Frye B., Scheinthal S., Kemarskaya T., Pruchno R. (2007). Tai chi and low impact exercise: effects on the physical functioning and psychological well-being of older people. *Journal of Applied Gerontology*.

[40] Innes K. E., Selfe T. K. (2012). The effects of a gentle yoga program on sleep, mood, and blood pressure in older women with restless legs syndrome (RLS): A preliminary randomized controlled trial. *Evidence-Based Complementary and Alternative Medicine*.

[41] Ong J. C., Manber R., Segal Z., Xia Y., Shapiro S., Wyatt J. K. (2014). A randomized controlled trial of mindfulness meditation for chronic insomnia. *SLEEP*.

[42] Newton K. M., Reed S. D., Guthrie K. A. (2014). Efficacy of yoga for vasomotor symptoms: a randomized controlled trial. *Menopause*.

[43] Afonso R. F., Hachul H., Kozasa E. H. (2012). Yoga decreases insomnia in postmenopausal women: A randomized clinical trial. *Menopause*.

[44] Köhn M., Persson Lundholm U., Bryngelsson I.-L., Anderzén-Carlsson A., Westerdahl E. (2013). Medical yoga for patients with stress-related symptoms and diagnoses in primary health care: A randomized controlled trial. *Evidence-Based Complementary and Alternative Medicine*.

[45] Liberati A., Altman D. G., Tetzlaff J. (2009). The PRISMA statement for reporting systematic reviews and meta-analyses of studies that evaluate healthcare interventions: explanation and elaboration. *British Medical Journal*.

[46] Buysse D. J., Reynolds C. F., Monk T. H., Berman S. R., Kupfer D. J. (1989). The Pittsburgh Sleep Quality Index: a new instrument for psychiatric practice and research. *Psychiatry Research*.

[47] Morin C. M., Belleville G., Bélanger L., Ivers H. (2011). The insomnia severity index: Psychometric indicators to detect insomnia cases and evaluate treatment response. *SLEEP*.

[48] Higgins J. P. T., Altman D. G., Gøtzsche P. C. (2011). The Cochrane Collaboration's tool for assessing risk of bias in randomised trials. *British Medical Journal*.

[49] Cohen J. (1988). *Statistical Power Analysis for the Behavioral Sciences*.

[50] Irwin M. R., Olmstead R., Carrillo C. (2017). Tai Chi Chih compared with cognitive behavioral therapy for the Treatment of Insomnia in Survivors of Breast Cancer: A randomized, partially blinded, noninferiority trial. *Journal of Clinical Oncology*.

[51] Wong S. Y., Zhang D., Li C. C. (2017). Comparing the effects of mindfulness-based cognitive therapy and sleep psycho-education with exercise on chronic insomnia: A randomised controlled trial. *Psychotherapy and Psychosomatics*.

[52] Johns S. A., Brown L. F., Beck-Coon K., Monahan P. O., Tong Y., Kroenke K. (2015). Randomized controlled pilot study of mindfulness-based stress reduction for persistently fatigued cancer survivors. *Psycho-Oncology*.

[53] Larkey L. K., Roe D. J., Weihs K. L. (2014). Randomized controlled trial of qigong/tai chi easy on cancer-related fatigue in breast cancer survivors. *Annals of Behavioral Medicine*.

[54] McQuade J. L., Prinsloo S., Chang D. Z. (2017). Qigong/tai chi for sleep and fatigue in prostate cancer patients undergoing radiotherapy: a randomized controlled trial. *Psycho-Oncology*.

[55] Lengacher C. A., Reich R. R., Paterson C. L. (2015). The effects of mindfulness-based stress reduction on objective and subjective sleep parameters in women with breast cancer: A randomized controlled trial. *Psycho-Oncology*.

[56] Zhang R., Yin J., Zhou Y. (2017). Effects of mindfulness-based psychological care on mood and sleep of leukemia patients in chemotherapy. *International Journal of Nursing Sciences*.

[57] Gong H., Ni C.-X., Liu Y.-Z. (2016). Mindfulness meditation for insomnia: A meta-analysis of randomized controlled trials. *Journal of Psychosomatic Research*.

[58] Boettcher J., Åström V., Påhlsson D., Schenström O., Andersson G., Carlbring P. (2014). Internet-based mindfulness treatment for anxiety disorders: A randomized controlled trial. *Behavior Therapy*.

[59] Garland S. N., Rouleau C. R., Campbell T., Samuels C., Carlson L. E. (2015). The comparative impact of mindfulness-based cancer recovery (MBCR) and cognitive behavior therapy for insomnia (CBT-I) on sleep and mindfulness in cancer patients. *Explore: The Journal of Science and Healing*.

[60] Nguyen M. H., Kruse A. (2012). A randomized controlled trial of Tai chi for balance, sleep quality and cognitive performance in elderly Vietnamese. *Clinical Interventions in Aging*.

[61] Irwin M. R., Olmstead R., Carrillo C. (2014). Cognitive behavioral therapy vs. Tai Chi for late life insomnia and inflammatory risk: a randomized controlled comparative efficacy trial. *SLEEP*.

[62] Sarris J., Byrne G. J. (2011). A systematic review of insomnia and complementary medicine. *Sleep Medicine Reviews*.

[63] Maddali Bongi S., Paoletti G., Calà M., Del Rosso A., El Aoufy K., Mikhaylova S. (2016). Efficacy of rehabilitation with Tai Ji Quan in an Italian cohort of patients with Fibromyalgia Syndrome. *Complementary Therapies in Clinical Practice*.

[64] Irwin M. R., Cole J. C., Nicassio P. M. (2006). Comparative meta-analysis of behavioral interventions for insomnia and their efficacy in middle-aged adults and in older adults 55+ years of age. *Health Psychology*.

[65] Li F., Fisher K. J., Harmer P., Irbe D., Tearse R. G., Weimer C. (2004). Tai chi and self-rated quality of sleep and daytime sleepiness in older adults: a randomized controlled trial. *Journal of the American Geriatrics Society*.

[66] Jahnke R. (2002). *The Healing Promise of Qi: Creating Extraordinary Wellness Through Qigong and Tai Chi*.

[67] Cohen K. S. (1999). *The Way of Qigong: The Art and Science of Chinese Energy Healing*.

[68] Lynch M., Sawynok J., Hiew C., Marcon D. (2012). A randomized controlled trial of qigong for fibromyalgia. *Arthritis Research & Therapy*.

[69] Chen M.-C., Liu H.-E., Huang H.-Y., Chiou A.-F. (2012). The effect of a simple traditional exercise programme (Baduanjin exercise) on sleep quality of older adults: a randomized controlled trial. *International Journal of Nursing Studies*.

[70] Chan J. S. M., Ho R. T. H., Chung K.-f. (2014). Qigong exercise alleviates fatigue, anxiety, and depressive symptoms, improves sleep quality, and shortens sleep latency in persons with chronic fatigue syndrome-like illness. *Evidence-Based Complementary and Alternative Medicine*.

[71] Chen Z., Meng Z., Milbury K. (2013). Qigong improves quality of life in women undergoing radiotherapy for breast cancer: results of a randomized controlled trial. *Cancer*.

[72] Telles S., Naveen K. V. (1997). Yoga for rehabilitation: An overview. *Indian Journal of Medical Sciences*.

[73] Dhruva A., Miaskowski C., Abrams D. (2012). Yoga breathing for cancer chemotherapy-associated symptoms and quality of life: results of a pilot randomized controlled trial. *The Journal of Alternative and Complementary Medicine*.

[74] Jindani F., Turner N., Khalsa S. B. S. (2015). A yoga intervention for posttraumatic stress: A preliminary randomized control trial. *Evidence-Based Complementary and Alternative Medicine*.

[75] Mustian K. M., Sprod L. K., Janelsins M. (2013). Multicenter, randomized controlled trial of yoga for sleep quality among cancer survivors. *Journal of Clinical Oncology*.

[76] Chen K.-M., Chen M.-H., Chao H.-C., Hung H.-M., Lin H.-S., Li C.-H. (2009). Sleep quality, depression state, and health status of older adults after silver yoga exercises: cluster randomized trial. *International Journal of Nursing Studies*.

[77] Cheung C., Wyman J. F., Resnick B., Savik K. (2014). Yoga for managing knee osteoarthritis in older women: a pilot randomized controlled trial. *BMC Complementary and Alternative Medicine*.

[78] Chandwani K. D., Perkins G., Nagendra H. R. (2014). Randomized, controlled trial of yoga in women with breast cancer undergoing radiotherapy. *Journal of Clinical Oncology*.

[79] Britton W. B., Haynes P. L., Fridel K. W., Bootzin R. R. (2010). Polysomnographic and subjective profiles of sleep continuity before and after mindfulness-based cognitive therapy in partially remitted depression. *Psychosomatic Medicine*.

[80] Kozasa E. H., Hachul H., Monson C. (2010). Mind-body interventions for the treatment of insomnia: A review. *Revista Brasileira de Psiquiatria*.

[81] Yang P.-Y., Ho K.-H., Chen H.-C., Chien M.-Y. (2012). Exercise training improves sleep quality in middle-aged and older adults with sleep problems: A systematic review. *Journal of Physiotherapy*.

[82] Buchanan D. T., Landis C. A., Hohensee C. (2017). Effects of yoga and aerobic exercise on actigraphic sleep parameters in menopausal women with hot flashes. *Journal of Clinical Sleep Medicine*.

[83] Cohen L., Warneke C., Fouladi R. T., Rodriguez M. A., Chaoul-Reich A. (2004). Psychological adjustment and sleep quality in a randomized trial of the effects of a Tibetan yoga intervention in patients with lymphoma. *Cancer*.

